# Utility of frozen section analysis for fungal organisms in soft tissue wound debridement margin determination

**DOI:** 10.1186/s13000-015-0423-9

**Published:** 2015-10-15

**Authors:** Nives Zimmermann, Matthew C Hagen, Jason J Schrager, Renee S Hebbeler-Clark, Sreeharsha Masineni

**Affiliations:** Department of Pathology and Laboratory Medicine, University of Cincinnati College of Medicine, Cincinnati, Ohio; Department of Surgery, University of Cincinnati College of Medicine, Cincinnati, Ohio; Department of Medicine, University of Cincinnati College of Medicine, Cincinnati, Ohio; Cincinnati Children’s Hospital Medical Center, Cincinnati, Ohio

**Keywords:** Fungi, Frozen section, Margin, Wound

## Abstract

**Background:**

Zygomycetes cause different patterns of infection in immunosuppressed individuals, including sino-orbito-cerebral, pulmonary, skin/soft tissue infection and disseminated disease. Infections with Zygomycetes have a high mortality rate, even with prompt treatment, which includes anti-fungal agents and surgical debridement. In some centers, clear margins are monitored by serial frozen sections, but there are no specific guidelines for the use of frozen sections during surgical debridement. Studies in fungal rhinosinusitis found 62.5–85 % sensitivity of frozen section analysis in margin assessment. However, the utility of frozen section analysis for margin evaluation in debridement of skin/soft tissue infection has not been published.

**Methods:**

We present a case of zygomycosis of decubitus ulcers in which we assessed statistical measures of performance of frozen section analysis for presence of fungal organisms on the margin, compared with formalin-fixed paraffin embedded (FFPE) sections as gold standard. A total of 33 specimens (94 blocks) were sectioned, stained with H&E and evaluated by both frozen and FFPE analysis. Negative interpretations were confirmed by Gomori methenamine silver stain on FFPE sections.

**Results:**

H&E staining of frozen sections had 68.4 % sensitivity and 100 % specificity for assessing margins clear of fungal organisms. The negative and positive predictive values were 70.0 % and 100 %, respectively. Using presence of acute inflammation and necrosis as markers of fungal infection improved sensitivity (100 %) at the expense of specificity (42.9 %).

**Conclusion:**

Use of intraoperative assessment of skin and soft tissue margins for fungal infection is a valuable tool in the management of skin and soft tissue fungal infection treatment.

## Background

Invasive fungal infections are an important cause of morbidity and mortality in immunocompromised hosts. While invasive candidiasis and invasive aspergillosis still account for the majority of these infections, zygomycetes also cause significant proportion of invasive fungal infections [1]. Zygomycosis refers to infections caused by fungi in the Zygomycota phylum, which includes pathogens such as *Mucor, Apophysomyces, Rhizomucor, Rhizopus and Absidia*. These infections occur with greater frequency in immunosuppressed patients with underlying diseases, such as diabetes and malignancy, but have also been described in previously healthy patients (reviewed in [2]). The infection most commonly involves sinuses (39 %), lung (24 %) and skin (19 %). The location of infection and likelihood of dissemination are also influenced by underlying clinical conditions. In a large meta analysis of zygomycosis cases (47 % *Rhizopus* species, 18 % *Mucor* species), the majority of patients with malignancy were found to have pulmonary infection (60 %), while those with diabetes had rhino-cerebral disease (43 %) [2]. Importantly, these infections have high mortality rates, which can be significantly influenced by the site of infection: 96 % in patients with disseminated disease, 76 % with pulmonary infections, and 31 % with cutaneous infections [2]. Patient outcomes are also significantly affected by treatment [2, 3]. Survival rates are: 3 % in untreated cases, 61 % and 57 % for patients treated with antifungals or surgery alone, respectively, and 70 % for patients treated with both antifungal agents and surgery [2]. Thus, optimal treatment for invasive zygomycosis is multi-modal, and includes antifungal agents, surgical debridement, and correction of underlying condition predisposing to the disease [4, 5].

There are reports in the literature of cases where surgical margins were evaluated by frozen sections intraoperatively [6–8]. Mathews et al. and Weinberg et al. describe cases of *Apophysomyces elegans* infection in previously healthy patients following C-section and brown recluse spider bite, respectively [6, 7]. In both cases, patients survived following a prolonged course of amphotericin B and multiple surgical debridements using frozen section analysis for margin assessment.

Intraoperative margin assessment is especially useful during rhino-sino-orbital fungal infections when delicate/vital structures, such as the orbit, could be spared if uninvolved. Case series on the role of frozen section in acute fungal sinusitis by Taxy et al. [9] and Ghadiali et al. [10] have shown 62.5 and 84 % sensitivity, respectively. The study by Ghadiali et al. [10] involved 20 patients with fungal rhinosunisitis over a 12 year period, 11 of which were infected with *Mucor* species, and 9 with *Aspergillus*. In a subgroup (6 patients; 1 with *Aspergillus* and 5 with *Mucor;* 30 slides total), frozen sections were used to assess margin status during surgical debridement. Using permanent section as the gold standard, the sensitivity (on a “per slide” basis) was 84 % and specificity was 100 %. The outcome of patients was not reported in this study.

The study by Taxy et al. [9] involved 8 patients with acute fungal sinusitis (including *Mucor, Aspergillus flavus, niger* and/or *fumigatus, Fusarium* and *Alternaria*) with both frozen and permanent sections, and in 5 of those cases fungal organisms were seen on frozen section. In two of the cases, fungal organisms were not seen on H&E-stained permanent sections either, and required special staining (methenamine). One case was negative in both frozen and permanent staining of frozen blocks, but positive on non-frozen tissue. Thus, the sensitivity (on a “per case” basis) of frozen sections for determination of margin status by frozen sections in this study was 62.5 %. Despite aggressive management, none of the patients survived.

However, no studies assessed the utility of intraoperative margin assessment for fungal infections of skin and soft tissue, where more liberal margins can be taken. Furthermore, in contrast to head and neck specimens, skin/soft tissue is technically more challenging due to increased adipose tissues and the surface area needing assessment could be much larger. These factors can significantly affect the performance of frozen section analysis for margin assessment.

## Methods

A 51-year-old female with past medical history of Stevens-Johnson syndrome (eye, s/p corneal transplants requiring immunosuppression with high-dose steroids and mycophenolate) and hypertension was admitted for management of pulmonary embolus and Legionella pneumonia. Following a complicated inpatient course, which included acute respiratory failure with acute respiratory distress syndrome (ARDS) requiring extracorporeal membrane oxygenation (ECMO), *Clostridium difficile* colitis, and steroid-induced hyperglycemia, she developed decubitus ulcers on her back and neck. Wound culture identified *Rhizopus microsporus var. rhizopodiformis*. The patient was started on IV liposomal amphotericin B and surgical wound debridement was performed. Margins were assessed by frozen section analysis and debridement continued until margins were confirmed clear. This took three separate surgical debridements over 4 days (days 1, 2 and 4 post wound culture organism identification). After 16 days of systemic liposomal amphotericin B, she was bridged to posaconazole for a total of 6 weeks of antifungal agents. The patient has been free of fungal infection since (>9 months of follow up), and the wounds have healed.

Soft tissue specimens were received fresh and the surgical margin was inked, shaved and submitted *en face* in the majority of specimens. Tissue was frozen in OCT, sectioned at 5–7 μm, and stained with H&E. Slides were microscopically assessed for the presence of branching non-septate hyphae. Presence of fungal elements anywhere on the *en face* slides was considered a positive margin, the surgeons were notified and the margin was reexcised. In a few initial specimens, the tissue margin was submitted perpendicular, and presence of fungal elements on ink was considered positive margin. Following pathological determination of frozen section, the tissue was fixed in formalin, processed by routine processing, embedded in paraffin, cut and stained with H&E for FFPE section analysis. In select cases, additional slides were stained with Gomori methenamine silver stain to highlight fungal elements. All slides were rereviewed for the research study and data presented are from this rereview. There was only one case of interpretation error, where margin was called negative for clinical purposes (on both frozen and FFPE), but fungal elements were found on rereview of the same slides (both frozen and FFPE).

Both slides (frozen and FFPE) were scored for presence of fungal elements and acute inflammation/necrosis. Data were entered into a truth table, with frozen section analysis as test and FFPE section as gold standard. Sensitivity, specificity, negative and positive predictive value were calculated. We calculated the performance on a “per specimen” basis because once a positive margin was identified on a slide, the margin was reported as positive and the remainder of the slides from the specimen were not analyzed.

This study has been reviewed by the University of Cincinnati Institutional Review Board and deemed “not human subjects research”.

## Results

**Fig. 1:**
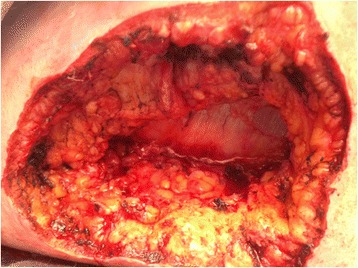
Representative gross image of one of the wounds

**Fig. 2:**
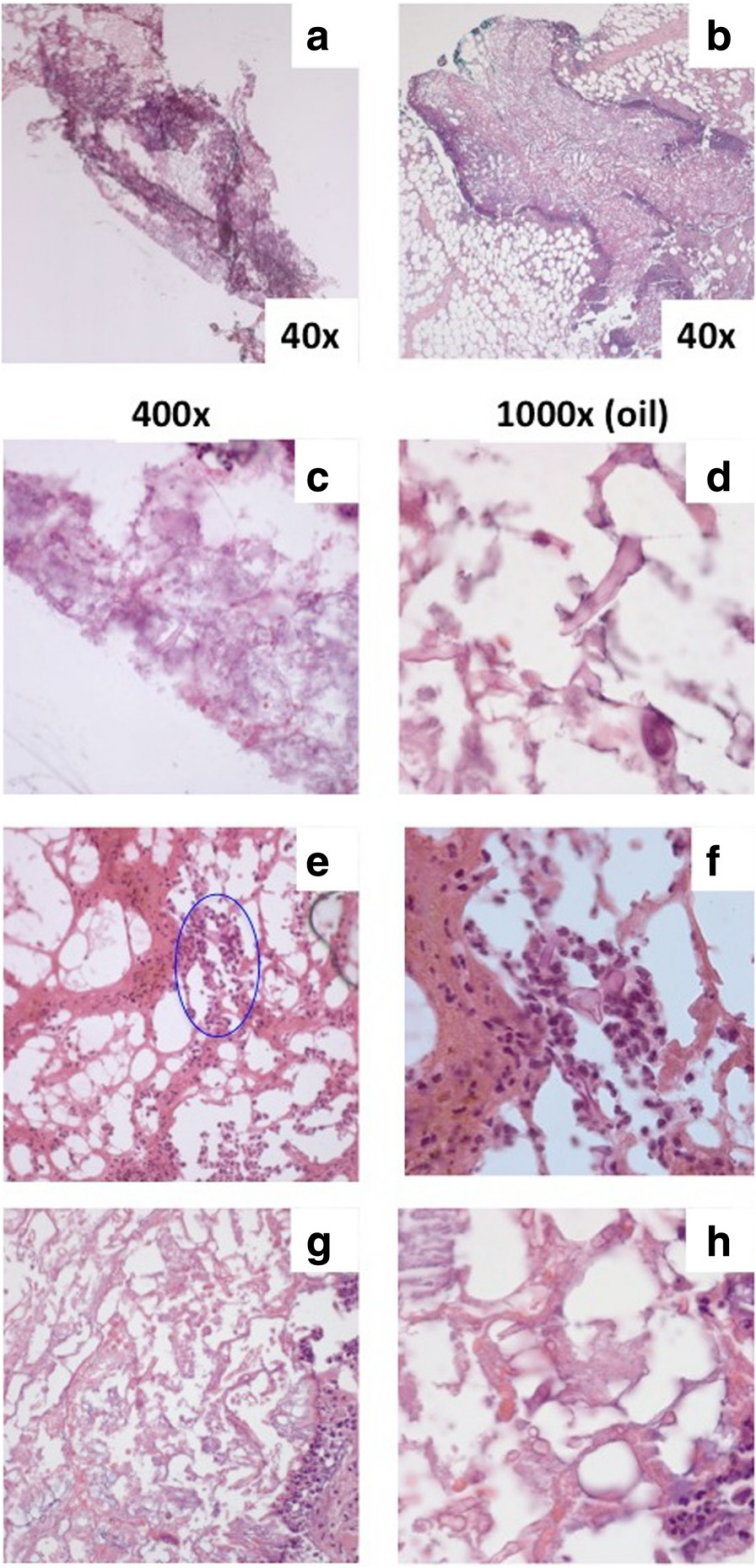
Representative images of fungal organisms on H&E stained frozen section (**a**, **c**-**f**) and FFPE section (**b**, **g**-**h**). Images taken at 40x magnification (**a** and **b**) demonstrate necrosis and inflammation, which were the areas most likely to contain fungal organisms. Panels **a**-**d** and **g**-**h** show images from slides with abundant organisms, while panels **e**-**f** are from slides with scarce fungal organisms with potential for false negative interpretation

The patient had three decubitus ulcers, two on the back and one on the neck (Fig. 1). She underwent three surgical debridement procedures. The first procedure had 26 specimens submitted for intraoperative consultation. Due to extensive length of the procedure (>9 h), the surgery was ended and resumed the next day when one additional margin specimen was submitted. Once FFPE section analysis demonstrated positive margins, which were missed at frozen section analysis, a third surgery was performed with 6 additional specimens analyzed intraoperatively. The average turnaround time was 17 min per specimen. Altogether, we compared frozen to FFPE sections in 33 specimens. The specimens had an average of 2.85 +/−1.66 (range 1–7) blocks/specimen for a total of 94 blocks that were analyzed by both frozen and FFPE section analysis. On FFPE section analysis, suspicious negative margins were confirmed with GMS special staining for fungal organisms, which failed to identify any organisms missed by H&E staining. Thus, analysis in this manuscript focused on determination of fungal organisms by H&E staining. Representative images of H&E-stained frozen and FFPE slides are shown in Fig. 2. Fungal elements were abundant in some frozen sections (example in Fig. 2a, c-d), but extremely rare in others (example in Fig. 2e-f). There was significant acute inflammation and necrosis present in the tissue, which highlighted areas more likely to contain fungal elements. However, presence of necrosis also made detection of fungal elements harder as fungal hyphae blended in with the pink strands of necrotic tissue. FFPE slides had improved tissue integrity (Fig. 2b compared with 2a which is frozen slide of same tissue block; and Fig. 2g-h at higher magnification, compared with 2c-d which are frozen slide of same tissue block), as well as ability to stain with Gomori methenamine silver stain to highlight fungal elements (data not shown).

Of the 33 specimens analyzed, 13 were positive for fungal elements on frozen section analysis, and all of those were confirmed on FFPE sections. In contrast, of the 20 specimens, which were called negative on frozen section analysis, 6 revealed fungal elements on FFPE. Thus, while the specificity is 100 %, the sensitivity of frozen section analysis is 68.4 %. The negative predictive value (NPV) and positive predictive value (PPV) is 70.0 % and 100 %, respectively (Table 1A).

**Table 1:** Statistical measures of performance of frozen section analysis for margin assessment during wound debridement surgery

A.		Fungal organisms on FFPE		
		yes	no	
Fungal organisms on frozen section	positive	13	0	13
	negative	6	14	20
		19	14	33
B.		Fungal organisms on FFPE		
		yes	no	
Inflammation/ necrosis on frozen section	positive	19	8	27
	negative	0	6	6
		19	14	33

Criteria used to call a margin positive were presence of fungalorganisms (in A) or presence of inflammation/necrosis (in B). Presence of fungal organisms of permanent sections was used as gold standard in both cases

Due to presence of abundant acute inflammation and necrosis as harbinger of fungal elements, we hypothesized that calling a margin positive based on presence of inflammation/necrosis alone would increase the sensitivity. Indeed, sensitivity and negative predictive value increased to 100 %; however, this lead to decreased specificity of 42.9 % and positive predictive value of 70.4 % (Table 1B).

## Discussion

We report a case of invasive fungal infection of decubitus ulcers in an immunosuppressed patient, treated with combination of surgical debridement and antifungal therapy. Margins were assessed for fungal elements by intraoperative analysis of frozen section slides, and later confirmed by FFPE sections. This allowed us to assess statistical measures of performance of the frozen section analysis.

The sensitivity in our case (68.4 %) was comparable to that published previously for head and neck fungal infection [9, 10]. A significant false negative rate is likely attributable to sampling bias, technical challenges of sectioning fatty tissue, frozen artifacts, and inconspicuousness of fungal organisms among other structures in necrotic tissue on H&E stained slides. Sampling bias could be due to FFPE sections being deeper, and thus further away from the true margin (in case of *en face* margins); however, this is likely still clinically significant as a positive margin. To over come the technical challenges of sectioning of fatty tissue and frozen artifact, one may consider using touch imprints or tissue surface scraping instead of frozen sectioning. Future studies should compare if this approach improves sensitivity. Detection of fungal elements may be improved with rapid fungal stains of frozen sections (such as rapid Romanowski stain, [9] or Gram staining [11] of tissue). However, when we attempted to retrospectively stain the FFPE tissues with above stains along with H&E, neither rapid Romanowski stain nor Gram stain improved detection of fungal organisms compared to H&E (data not shown).

Alternatively, instead of immediate intraoperative margin assessment, team members should consider improving accuracy of margin assessment with formalin fixation (leading to better tissue integrity), as well as special stains for fungal organisms, such as GMS (leading to improved detection of fungal elements). As such, team members need to discuss whether immediate results with potential false negatives are beneficial compared with more reliable but delayed results. Factors such as ability to repeat margin excision in a few days and proximity to vital structures should be considered. Another alternative to consider is rapid processing which may provide turnaround time in-between intraoperative consultation and routine FFPE processing. In addition to improving accuracy, considering alternatives to intraoperative consultation is important from the standpoint of time to results. Standard maximum turnaround time at our institution for frozen section analysis is 20 min per specimen. For instance, the initial surgery in our case consisted of 29 specimens (85 blocks total) submitted for margin assessment. Pathology assessment became the rate-limiting step causing the surgery to last more than 9 h. Prolonging surgery can have significant detrimental outcomes for the patient as it prolongs the time under anesthesia and associated risks. This is especially true in patients with invasive fungal infections who can be quite unstable at the start of the operation. Thus, anticipated time for intraoperative results should be calculated based on the wound size, and logistical approaches to increase speed without compromising quality should be considered. For instance, the number of personnel, available microtomes and other hardware could be optimized. In summary, improving time to results and accuracy of results would drive decisions to assess margins intra- or post-operatively, and if the former, a number of steps could be taken to improve patient’s outcome by improving speed and accuracy.

Furthermore, team members should discuss whether decreasing false negative calls, at the expense of increasing false positives, may be desirable and can be achieved by using acute inflammation and necrosis as surrogate markers of fungal elements. For example, if there is ample distance to vital structures and repeat surgery is undesirable based on the patient’s clinical comorbidities, this may be a viable approach. However, false positives and a specificity of 42.9 % come with their own set of challenges. Creating a larger than necessary wound burden carries with it prolonged wound care needs, increased chance of secondary infection, increased debility (with decreased mobility both short- and long-term) and possibly increased intensive care unit days (with increased risk of nosocomial infections, delirium, and even mortality). As such, the balance of false positives and false negatives needs to be carefully considered and the approach for calling positive margins should be agreed upon by pathologist and surgeon.

Our study has its limitations. First, our findings are limited to a single patient. However, this is a rare scenario and thus there is currently limited experience. By sharing our experience and analyzing the multiple samples (total of 33 specimens), we hope to provide new knowledge, increasing the awareness about the utility of this approach and its limitations, thus aiding in future decision making. Also, an increase in reported cases will facilitate study of a broader spectrum of cases with different clinicopathological and microbiological characteristics. The second limitation is that our study does not address whether clear margins during debridement are indeed necessary for optimal clinical outcome, and whether debridement with margin assessment is superior to debridement guided by other measures (e.g. assessment of margin viability and lack of infection by gross inspection). Evidence for use of frozen sections in margin assessment of infected soft tissue debridement is lacking in the literature, and the approach of debridement for clear margins is chosen based on clinical assessment (for instance, trying to balance wound management with the fact that an immunocompromised patient will be unable to clear even a low load of infection) and gravity of the situation (known high mortality of the disease). We are aware of only one case series that described cases with and without debridement guided by frozen sections [10]; however, patient outcome was not reported in that study. The questions our study does address are the analytic specificity and sensitivity of frozen section use for margin assessment during infected soft tissue debridement, and how they are affected by using identification of fungal organisms versus inflammation/necrosis. This provides useful information to team members for informed decision-making.

In summary, our study provides statistical measures of performance for frozen section analysis of margins during debridement surgery for invasive fungal infections of soft tissue and also provides comparison of these measures for different criteria (fungal organisms versus inflammation/necrosis), which should be considered when making decisions on which approach to use in individual clinical circumstances.

## Conclusion

Use of intraoperative assessment of skin and soft tissue margins for fungal infection is a valuable tool in the management of skin and soft tissue fungal infection treatment. Whether presence of fungal elements or necrosis/acute inflammation is used as cutoff to call positive margins needs to be decided based on clinical scenario using sensitivity and specificity presented here.

## Abbreviations

ARDS: Acute respiratory distress syndrome
FFPE: Formalin-fixed, paraffin-embedded
NPV: Negative predictive value
PPV: Positive predictive value
